# Original salivary sex hormone data of naturally menstruating athletes and hormonal contraceptive users

**DOI:** 10.1136/bmjsem-2024-002078

**Published:** 2024-11-13

**Authors:** Alice Lafitte, Marine Dupuit, Tom Chassard, Kilian Barlier, Nolwenn Badier, Martine Duclos, Jean-François Toussaint, Juliana da Silva Antero

**Affiliations:** 1 Institute for Research in Medicine and Epidemiology of Sports (IRMES, EA7329), INSEP, Paris, France; 2 Department of Sport Medicine and Functional Explorations, University-Hospital (CHU), G. Montpied Hospital, Clermont-Ferrand, France; 3 UMR 1019, INRA, Clermont-Ferrand, France; 4 Université Paris Cité, Paris, France; 5 CIMS, Hôtel-Dieu, AP-HP, Paris, France

**Keywords:** Athlete, Endocrine, Female, Measurement

## Abstract

**Objectives:**

There is a lack of data on salivary sex hormones across the menstrual cycle (MC) or hormonal contraceptive (HC) cycle of elite athletes. We aimed to provide original data on salivary sex hormones (17β-estradiol, progesterone and free testosterone) in naturally menstruating female athletes with a regular cycle or irregular cycle and using combined HC. A secondary purpose was to compare these data with published data from the general population according to the menstrual status (MC or HC or irregularly menstruating).

**Methods:**

367 saliva tests were performed on 44 elite athletes during 6 months of follow-up to certify for cycle regularity. Athletes were grouped into regular MC, n=13; irregular MC, n=5; and HC, n=26. We compared salivary data of regular MC across six cycle phases (menses, mid-follicular, late follicular, early luteal, mid-luteal and late luteal phases) with published data from women with a similar MC or HC status from the general population.

**Results:**

We provided salivary original data according to six sub-phases among elite athletes with regular MC. HC athletes showed lower salivary sex hormonal levels, markedly after the first week of active HC compared with regular MC. Athletes with irregular cycles do not show a progesterone rise from the first half to the second half of the cycle (Δirregular=0.38 (1.90), a rise detectable within regular MC group ΔregularMC=2.86 (2.88)).

**Conclusions:**

We provided original data for salivary sex hormone levels in elite female athletes. These references may be valuable for research investigating MC or combined HC data, particularly in longitudinal follow-ups requiring repeated measurements.

**WHAT IS ALREADY KNOWN ON THIS TOPIC**

- 17β-estradiol, progesterone and testosterone levels fluctuate through the menstrual cycle, which may affect elite athletes’ training and wellness.
- There is a lack of robust research relying on hormonal measurements to identify menstrual cycle phases and their effect on elite athletes.
- A serum blood sample is the gold standard for sex-hormonal measurement, but it is invasive and may preclude repeated measures from being used in longitudinal studies.
- Hormonal contraception down-regulates endogenous sex hormone levels, such as 17β-estradiol and progesterone concentration.

**WHAT THIS STUDY ADDS**

- We provided original salivary sex hormone data range across six menstrual cycle phases in naturally menstruating athletes with regular and irregular cycles.
- Athletes with irregular cycles and athletes using hormonal contraception do not exhibit a rise in salivary progesterone in comparison with athletes with regular menstrual cycles.
- Athletes using hormonal contraception show lower levels of free testosterone compared with naturally menstruating athletes.

**HOW THIS STUDY MIGHT AFFECT RESEARCH OR PRACTICE**

- Determining baseline values for sex hormone levels in saliva with repeated measures across at least three menstrual cycles could offer a starting point for future studies on elite female athletes across the menstrual cycle or under the use of combined hormonal contraception.
- The salivary sex hormone concentrations range of naturally menstruating athletes with a regular menstrual cycle may be valuable for longitudinal studies requiring repeated measurements, benefiting from a less-invasive option.
- The differences observed in sex-hormonal levels among a regular cycle, irregular cycle and hormonal contraception may enlighten new hypotheses for investigating the effects of menstrual status or use of hormonal contraception on elite athletes.

## Introduction

 Female sex hormonal variations can characterise, in terms of timing and amplitude, the nature of the menstrual cycle.^1^ These hormones, including oestrogen and progesterone, fluctuate throughout the natural menstrual cycle (MC) or are downregulated during hormonal contraception (HC). The eumenorrhoeic cycle begins in menses when levels of oestrogen and progesterone are low. During the follicular phase (FP, lasting from day 1 to ovulation), oestrogens rise until they peak just before ovulation and decrease shortly after. During the luteal phase (LP, from ovulation to the next menses), progesterone is more abundantly produced: it peaks in the middle of the LP and drops at the end when, at the same time, oestrogens rise and fall again.^2^ Hormonal dysfunctions can occur during menstrual cycles, as especially exercising women show a high prevalence of menstrual disturbances.^3^

Not only do oestrogens and progesterone fluctuate, but also testosterone fluctuates and is produced in the ovaries. It is mainly bound to sex hormone-binding globulin (SHBG) and albumin. Only a small fraction (1–2%) circulates as nonprotein-bound free testosterone. Free testosterone only enters cells and exerts androgen activity.^4^ Several studies have described changes in free testosterone concentrations during MC.^5^ For Cook *et al*,^6^ testosterone concentrations rise from the LP to the ovulatory phase and fall during LP. Other authors found serum levels of total and free testosterone similar in FP and LP, with a slight pre-ovulatory increase^7^ concomitantly with 17β-estradiol fluctuations (*ie,* the most abundant oestrogen) in women with normal menses. Oestrogen and testosterone have anabolic action on the body,^8 9^ which could be linked to similar fluctuations in MC phases. Studies of testosterone levels have provided inconclusive and mixed results regarding systematic changes in testosterone throughout the cycle. Although methods and study designs differed between studies, sample sizes appeared small, and some studies used rough estimates to determine the cycle phase. Knowledge about free testosterone variations during MC seems insufficient.

Exogenous hormones in HC downregulate endogenous hormones, altering the normal functioning of the ovaries and endometrium. HC contains ethinylestradiol and progestins, which can be androgenic or anti-androgenic. The androgenic HC slightly counteracts the effects induced by the decreasing levels of testosterone in HC users, but the anti-androgenic reinforces them.^10^ Anti-androgenic progestins are often used, such as levonorgestrel and norethindrone, not only as a form of contraception but also to reduce androgenisation symptoms.^11^,^13^ Bioavailable testosterone, including free and albumin-bound testosterone (ie, testosterone not bound to SHBG), was significantly suppressed by different mechanisms depending on the progestin used.^11^ Some studies have shown higher free testosterone levels in natural MC women compared with HC users.^14 15^ Such studies, however, involved a single cycle, and questions also remained related to the precise phases when measurements were taken, as well as whether the salivary test would capture such differences.

The hormonal fluctuations throughout the MC and HC cycles have been suggested to have numerous psychological and physiological effects^16^,^20^ that could ultimately impact elite athletes’ performance^21^ or injury risk.^22^ Elite athletes had a high volume of daily intensive training, which can lead to hormone profiles that differ significantly from those of non-athletes and established reference ranges. While acute physical exercise has been shown to elevate levels of estradiol, testosterone and growth hormone,^23^ limited data exist on the influence of MC or HC phases on physical performance in elite athletes, and the quality of the available data is often insufficient. This scarcity can be attributed, in part, to challenges in accurately identifying and verifying menstrual cycle phases, which requires using the so-called three-step method.^24^ In addition, to ascertain any potential relation between hormonal phases and performance, longitudinal follow-ups requiring repeated measures of hormonal samples are essential.^21^ A blood serum test is the gold standard for collecting hormonal variations during MC.^25^ However, repeated measures of blood samples are invasive and hardly feasible on the field, especially with elite athletes.

Salivary testing is a simpler, more cost-effective and less-invasive alternative to blood or 24-hour urine sampling for longitudinal follow-up. A recent review summarised the findings from several studies comparing salivary- and blood-derived hormonal concentrations and concluded that the correlation coefficients of progesterone and 17β-estradiol are moderate to very high,^26^ and another recent study established a good correlation between salivary and capillary blood progesterone to profile the menstrual cycles of young professional soccer players,^27^ but larger validation studies should be conducted. However, there is also a lack of research on the salivary sex hormonal profile of elite athletes, particularly in characterising differences between regular and irregular MC and HC. MC phases are often subdivided into sub-phases.^28 29^ FP and LP are often divided into three subphases, ie, the early, mid and late sub-phases, yet there is a lack of reference values for each. The reference values provided by independent laboratories typically display wide ranges due to inter- and intra-individual physiological variations, even in cycle length, bleeding pattern and timing.^30^,^32^ Additionally, to our knowledge, there are no references for endogenous fluctuations of such hormones among elite athletes HC users.

The first purpose of this study was to provide original data on salivary sex hormones (17β-estradiol, progesterone and free testosterone) in naturally menstruating female athletes with a regular cycle or irregular cycle and using combined HC. A second purpose was to compare these data with published data from the general population and according to the menstrual status (MC or HC or irregularly menstruating).

## Methods

### Study design

Longitudinal study based on repeated measurements of a cohort of elite athletes.

### Participants

#### Athletes

Fifty-four elite athletes (Tiers 4–5^33^ according to the Participant Classification Framework), including 12 skiers, 9 cyclists, 10 rowers, 10 swimmers, 8 triathletes and 5 wrestlers, were informed and volunteered to participate in this study from February 2021 up to February 2024. In addition, their written informed consent was collected. They were asked to complete a preliminary questionnaire to collect general information (eg, age, body mass, height, training volume) and their gynaecological history (eg, menarche, cycle regularity, contraceptive methods). Twenty athletes had a natural MC (including two using copper-based intrauterine devices). Copper-based intrauterine devices only act on the endometrium and do not deliver synthetic hormones. A regular natural cycle can be found in some women using a copper intrauterine device. Twenty-three used combined HC and formed the HC group. Eleven used progestin-only HC (including oral contraception, ring and hormonal intrauterine devices; see online supplemental table 2) and were not included. Athletes were followed for 4 to 6 months to follow at least three complete cycles.^25^ The first 3 months were used to monitor whether the athlete had regular or irregular cycles among the MC group. A regular cycle was determined through a minimal 3 month follow-up. It was defined as a cycle-to-cycle length variation lower than 7 days^34^ and a cycle length between 21 and 35 days. Follow-up continued for at least two or three more cycles to confirm cycle regularity over the 6 month longitudinal follow-up. Naturally menstruating athletes not filling these requirements were regrouped into the MC irregular group.^25^ Yet, among athletes in the MC group, there might be undetected subtle menstrual disturbances, such as anovulatory cycle or LP inadequacy (LP defect). Athletes with secondary amenorrhoea (absence of menstrual bleeding for 3 or more consecutive months^35^) and athletes suffering from a medically detected chronic disease or syndrome (endometriosis or polycystic ovary syndrome) were excluded.

#### General population

##### Search strategy

The search focused on studies of women within general populations that directly investigated salivary sex hormonal concentration during MC. Participants defined as eumenorrhoeic women (as menstrual cycles lasting ≥21 days and ≤35 days giving rise to 9 or more consecutive periods per year, proof of ovulation, a correct hormonal profile and no use of HC 3 months before recruitment^25^) or naturally menstruating (as menstrual cycle lengths ≥21 days and ≤35 days, without confirmed ovulation) according to the methods of each article. Primary work was conducted in the PubMed, ResearchGate and ScienceDirect databases to identify eligible papers using a well-defined, priori-formulated search strategy. It was conducted using a Boolean approach following search terms: (“salivary” OR “salivary test” OR “saliva” OR “salivary profiles”) AND (“17β-estradiol” OR “estrogens” OR “progesterone” OR “testosterone OR “hormones” OR “sex hormones”) AND (“menstrual cycle” OR “menstrual cycle phase” OR “luteal phase” OR “follicular phase”). The final search was updated on 1 December 2023 without a limit on publication year. The search was completed using the references collected in the bibliography of included studies and overview articles.

##### Study selection

Studies that reported at least one salivary sex hormonal concentration (17β-estradiol, progesterone or free testosterone) related to MC in healthy women were included. We defined ‘healthy woman’ as an eumenorrhoeic or naturally menstruating woman with a regular MC, no OC-use or other hormonal agent within the past 6 months. No age restrictions were applied.

For 17β-estradiol and progesterone data, we excluded studies that did not perform saliva testing in the morning and those where participants were not fasting less than 8 hours, had not performed the test before breakfast, had not been fasting for ≥3 hour or had consumed caffeine/alcohol ≤12 hour. They could only drink water up to an hour before the test.

Due to the lack of studies, we used less strict inclusion criteria for free testosterone data. The inclusion criteria were extended to the time of salivary sampling. We included studies in which participants were not fasting during sampling. We also included studies where participants were instructed not to smoke, not to eat and not to drink fruit juice 1 hour before testing or alcohol 12 hours before, not to brush their teeth 45 min before collecting saliva.

### Data collection

#### Menstrual phases determination

Before the beginning of the follow-up, the protocol was presented to each athlete. After approval,^36 37^ each of them responded to a daily questionnaire on an online app to enter the first day of their menstruations for natural MC athletes or of the pill’s pause (or inactive phase) for HC athletes.

Based on cycle length and regularity, we estimated the most probable ovulation day based on a linear regression relying on data from more than 30 000 women with ovulation confirmed to establish FP and LP in the regular MC group.^38^ As described previously, FP and LP were divided into early, mid and late sub-phases.^37^

For HC athletes, cycles were divided into four phases: the pills’ pause and 3 weeks while taking a pill or receiving hormones (week 2, week 3 and week 4).

For irregular athletes, cycles were divided into their menstruation phase, and the rest of the cycle was called the no-menstruation phase.

#### Salivary test

Salivary samples of 17β-estradiol, progesterone and free testosterone were collected during follow-up: the saliva sampling period was carried out over one to two cycles after 2 to 3 months of previous follow-up for cycle regularity monitoring. Follow-up extended over two to three additional cycles to confirm cycle regularity for 6 months. For regular MC athletes, samples were collected on days 3, 8 and every 2 days until the end of the cycle. For the irregular MC group, samples were taken the same way as for the MC group, with a maximum of 12 consecutive tests. For HC athletes, two salivary tests were carried out during the first phase (ie, inactive phase) and three others over the following 3 weeks. This sampling method was based on the reliability demonstrated in our pilot study and supported by previous research using repeated salivary measurements^37 39^ under similar conditions that showed reproducible results.

17β-estradiol, progesterone and free testosterone concentrations were measured using a saliva collection kit (SaliCap Set, IBL International GmbH, Germany). Athletes were instructed to collect a saliva sample on waking, fasting and at least 30 min after drinking. They were also advised to rinse the oral cavity with water and not to chew gums just before saliva collection. A polypropylene straw was used to salivate the SaliCap. All sampling vials were properly labelled once the tube was half-filled with saliva (patient name, date, time). Athletes were instructed to store tubes in their fridge (between 2 and 8°C) until the expedition. They had to send their saliva sample within 48 hours (saliva samples could be stored at temperatures between 2 and 8°C for up to 4 weeks without affecting the validity of the test; if saliva samples were stored at −20°C and below, they could be kept for longer).^40^,^42^ The BIOAVENIR laboratory (Metz, France) performed the test analysis procedure following the appropriate protocol recommendations, using reagents from the German company Tecan, IBL International and luminescent immunoassay (IBL, Hamburg, Germany). The luminescence was measured on a Berthold Luminometer. Analytical sensitivities (CI 95%) were 0.3 pg.mL^−1^ (17β-estradiol), 2.4 pg.mL^−1^ (progesterone) and 1.8 pg.mL^−1^ (free testosterone). For estradiol, intra- and inter-assay coefficients were 6.4% and 12.8%, respectively. For progesterone, intra- and inter-assay coefficients were 7.7% and 5.3%, respectively. For free testosterone, intra- and inter-assay coefficients were 9.1% and 4.7%, respectively.

Hormonal analysis procedures are detailed in the online supplemental material.

### Data analyses

Z-score-based methods commonly used to identify atypical values in biological analyses^43^ were used to identify abnormal data. An observation was considered abnormal based on the test statistic*, T_n_*, calculated from the studentised residuals. Monte Carlo simulations were used to estimate the quantiles of the distribution of *T_n_*. These quantiles were then used as a threshold to detect outliers. An observation was considered abnormal based on the test statistic*, T_n_*, calculated from the studentised residuals (*T_n_* of *x_n_* higher than *c*, the distribution quantile of order 1 – α/2). All identified abnormal data were excluded from the study.

The analyses to meet the first objective involved calculating the hormonal means, SD, medians and two quartiles (bottom and upper) for each sub-phase of the MC and HC groups.

The analyses for the second objective were carried out by analysing the different hormone levels in each group (regular MC users, irregular MC users, HC users). Since the cycle phases do not have the same length between groups, we transformed the cycle length into a proportion of advancement in the cycle. We calculated the normalised time for each athlete’s cycle (0 corresponded to the first day of each cycle and one to the last). This proportion of advancement in the cycle has been split into two parts (first and second cycle half). The first part covers 1% to 49.99% of the cycle advancement, and the second covers 50% to 100%. For each group, a non-parametric Loess regression was performed to explain the progress of the hormonal cycle as a function of the athlete group. We calculated the average values estimated by the model at each moment of the cycle as well as its CI. We calculated a hormonal variation index (Δ) between the two parts of the cycle advancement of each group of athletes. This index was determined by subtracting the hormonal levels in the second phase from those in the first phase and then dividing the difference by the hormonal levels in the first phase. A positive Δ indicates an increase in hormonal levels between the two phases, a negative Δ reflects a decrease and a Δ near zero suggests stable hormonal levels throughout the cycle. Wilcoxon’s test was used to compare the salivary hormone levels of the MC and HC groups in the first part and then in the second part of the cycle and to compare their Δ. The same method was used to compare regular and irregular MC groups.

The salivary data from the MC group and those from the general population were compared with meet the second objective. From the results of previous articles, we calculated the average hormone levels for each of the six cycles of sub-phases. The Wilcoxon test compared salivary hormone levels between the general population and regular MC athletes. A comparison was not conducted for HC athletes, as there is no existing reference for salivary hormonal variations among the general population, but means and SD were calculated. Wilcoxon Test and Kruskal–Wallis test were used to compare the hormonal data of HC athletes across cycle sub-phases.

R software was used for all analyses, and the significance threshold was set as α=0.05.

### Research ethics and data security

The conducted investigations adhered to the code of ethics outlined by the World Medical Association (Declaration of Helsinki) and received approval from the Institutional Ethics Committee (IRB00012476-2023-06-11-275). The data collection process complied with the General Data Protection Regulation (2016/679) implemented in the European Union and received a certificate of compliance from the *Commission Nationale Informatique et Libertés* (CNIL - 2 221 532 v0).

### Patient and public involvement

This study was designed to answer the coach and athletes’ questions regarding the effect of the menstrual cycle or the contraceptive hormones in their training monitoring. We co-created the protocol to adapt it to their training set.

### Statement on equity, diversity and inclusion

Our research and author team consists of five female authors and three male authors from France, all interested in female athlete health. This team comprises junior, mid-career and senior researchers from different disciplines, including biostatistics, sports medicine, epidemiology, endocrinology and physiology. The study focused exclusively on female elite athletes, given our specific aim of examining the hormonal fluctuations of this population depending on the menstrual profile.

## Results

### Participants

Among 57 athletes, 44 met the inclusion criteria and completed the study. Four athletes were excluded. One athlete was suffering from amenorrhoea and had changed contraception at the time of sampling. A second in amenorrhoea had only one cycle during her follow-up, but this never ended because the follow-up was stopped beforehand. One athlete had polycystic ovary syndrome and was amenorrhoeic; she never menstruated during follow-up. Another athlete suffered from endometriosis and was on continuous contraception. Athletes included were 17–38 years old (25.4±8.2, median=24) and trained 6 to 7 days a week. On average, 4.5 full cycles per athlete were followed. The regular MC group comprised 13 athletes (including one using copper-based intrauterine devices), the HC group of 21 athletes (15 using monophasic pills, 2 biphasic pills and 4 triphasic pills) and the irregular group of five athletes. 333 saliva tests were analysed (130 from regular MC, 159 from HC and 44 from athletes in the irregular group). An average of 10 (± 2) saliva tests were carried out per athlete in the regular MC, 7 (± 4) tests on HC and 8 (± 3) tests on irregular athletes.

### Natural MC group

For the natural regular MC group, 17β-estradiol and progesterone levels significantly vary during cycle phases (p=0.002, p=0.001, respectively). Free testosterone varies according to the phases of the regular natural cycle, but there are no significant differences between these phases for this group.

For the natural irregular MC group, 17β-estradiol and progesterone levels do not vary significantly between the menstruation phase and no-menstruations (p=0.106, p=0.091, respectively). Salivary-free testosterone levels in the menstrual phase are significantly different in the no-menstruation phase (p=0.034).

### HC group

**Figure 1:**
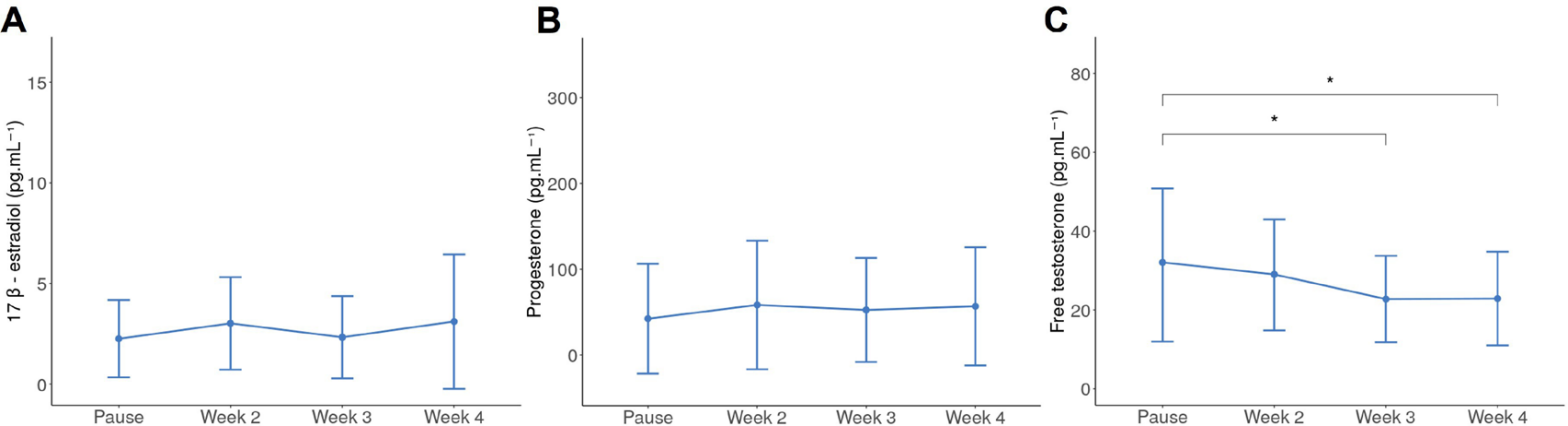
Mean and SD of salivary hormone levels (pg.mL^−1^) HC athletes during the sub-phases of the cycle. (**A**) 17β-estradiol, (**B**) Progesterone, (**C**) free testosterone; *p<0.05

There was only a trend for the HC group, but no significant differences between the four phases determined for this group regarding 17β-estradiol and progesterone salivary levels (p=0.07 and p=0.07, respectively) (figure 1A,B). Salivary-free testosterone levels in the pill’s pause (or inactive phase) are significantly different in the last 2 weeks of HC (p=0.03, figure 1C). Table 1 lists the salivary values during the sub-phases of each cycle type for natural, regular or irregular MC and HC athletes for each hormone.

**Table 1:** Salivary values of 17β-estradiol, progesterone and free testosterone during the cycle sub-phases of natural regular, irregular MC groups and HC group of elite athletes

Sub-phases	Salivary 17β-estradiol (pg.mL^-1^)			Salivary progesterone (pg.mL^-1^)			Salivary-free testosterone (pg.mL^-1^)		
	Mean±SD	Median	Q1;Q3	Mean±SD	Median	Q1;Q3	Mean±SD	Median	Q1;Q3
Natural regular MC Group									
Menses	3.05±1.65	2.06	1.56; 3.09	39.42±36.49	27.20	20.70; 45.60	28.12±24.35	29.43	14.45; 35.64
MFP	3.06±1.81	2.50	1.72; 3.79	31.18±24.44	30.75	16.75; 38.50	24.60±16.66	27.50	15.70; 28.75
LFP	5.37±4.54	3.56	1.95; 8.08	44.21±55.54	32.00	17.70; 44.90	33.11±18.58	35.01	24.65; 43.20
ELP	4.46±2.22	3.48	3.12; 5.62	119.50±153.00	90.10	32.02; 158.28	28.11±18.48	24.28	17.24; 37.38
MLP	6.50±2.69	5.84	4.81; 7.62	171.81±255.81	130.35	53.42; 208.18	35.14±22.81	41.9	16.20; 49.10
LLP	4.57±1.15	5.15	3.08; 5.25	83.01±46.42	81.90	63.78; 89.42	27.43±18.26	25.69	19.08; 31.34
HC group									
Inactive phase	2.26±1.99	1.47	0.97; 3.26	23.10±69.09	42.24	17.30; 40.60	31.39±20.88	26.30	16.70; 44.15
Week 2	2.83±2.15	2.33	1.54; 3.82	33.15±87.02	58.20	19.27; 54.90	28.88±17.37	25.80	22.24; 31.56
Week 3	2.33±2.17	1.63	0.90; 2.62	32.00±75.24	52.33	23.37; 59.42	22.75±10.10	19.69	13.90; 31.38
Week 4	2.77±3.33	1.66	1.37; 3.13	32.40±68.96	56.65	19.58; 54.88	22.88±11.88	17.80	13.90; 30.97
Irregular MC group									
Menses	3.59±2.68	2.89	1.59; 4.49	33.27±19.69	29.40	22.40; 34.00	22.10±11.35	17.80	14.30; 32.20
No menses	5.14±3.54	4.43	2.35; 7.03	45.7±35.26	36.10	23.50; 52.45	30.74±18.23	27.5	18.22; 40.20

ELP, Early luteal phase; LFP, Late follicular phase; LLP, Late luteal phase; MFP, Mid-follicular phase; MLP, Mid-luteal phase; Q1, Bottom quartile; Q3, Upper quartile

### Natural regular MC vs HC groups

**Figure 2:**
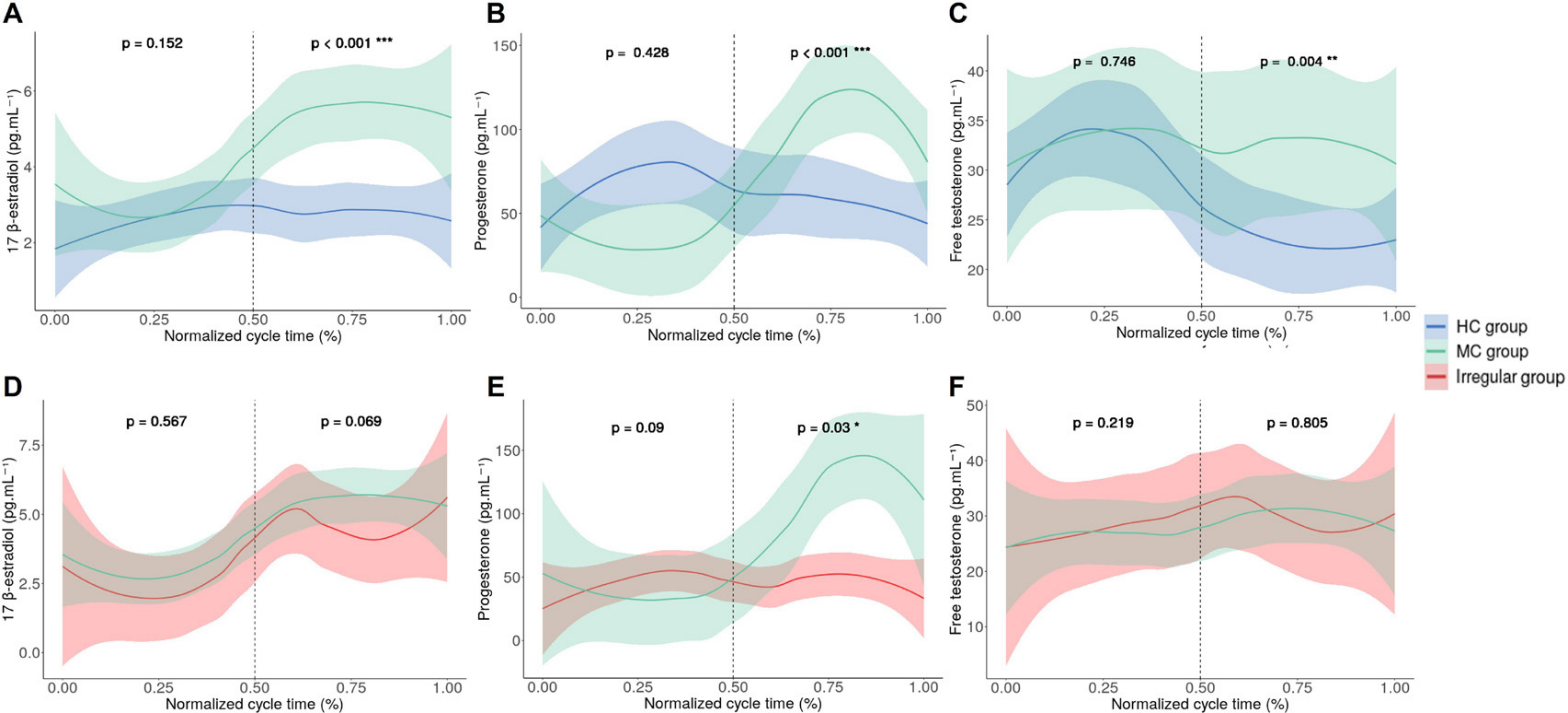
Mean and estimated CIs of salivary hormone concentration of athlete groups across the normalised hormonal cycle among athletes as a function of % cycle advancement. The dotted lines represent the division in two of the progress of the cycles. The MC group is green, the HC group is blue and the irregular group is red. (**A**): 17-β estradiol MC group vs HC group. (**B**): Progesterone MC group vs HC group. (C): Free testosterone MC group vs HC group. (**D**): 17-β estradiol MC group vs irregular group. (**E**): Progesterone MC group vs irregular group. (**F**): Free testosterone MC group vs irregular group. *p<0.05, **p<0.005, ***p<0.05

Salivary 17β-estradiol and free testosterone index of variation do not differ between these groups. Natural regular MC and HC groups have significant differences in salivary 17β-estradiol (figure 2A), progesterone (figure 2B) and free testosterone (figure 2C) in the second part, from 50% to 100% of the cycle advancement. Table 2 shows the different indices of variation of hormones between the two halves of the cycle for each group. The progesterone variation index significantly differed between the natural regular MC and HC groups.

**Table 2:** Hormonal variation index (Δ) between the two parts of the cycle advancement of each group of athletes

	ΔregularMC	ΔHC	Δirregular
17β-estradiol (pg.mL^−1^)	1.51 (0.48)	0.9 (0.30)	1.68 (0.30)
Progesterone (pg.mL^−1^)	2.86 (2.88)	0.81 (0.51)*	0.38 (1.90)†
Free testosterone (pg.mL^−1^)	0.97 (0.68)	−0.72 (0.51)	1.12 (1.62)

Values are Δ (SD).

* p < 0.05 compared ΔregularMC and or ΔHC.

† p < 0.05 compared ΔregularMC and Δirregular.

‡ p < 0.05 compared ΔHC and Δirregular.

### Natural regular vs irregular MC groups

Regular and irregular MC groups significantly differ in salivary progesterone (figure 2E) in the second part of the cycle advancement. Salivary 17β-estradiol and free testosterone levels do not differ between these groups (figure 2D,F). The index of variation for progesterone was significantly different between the natural regular MC and irregular groups (table 2).

### General population

#### Study selection

**Figure 3:**
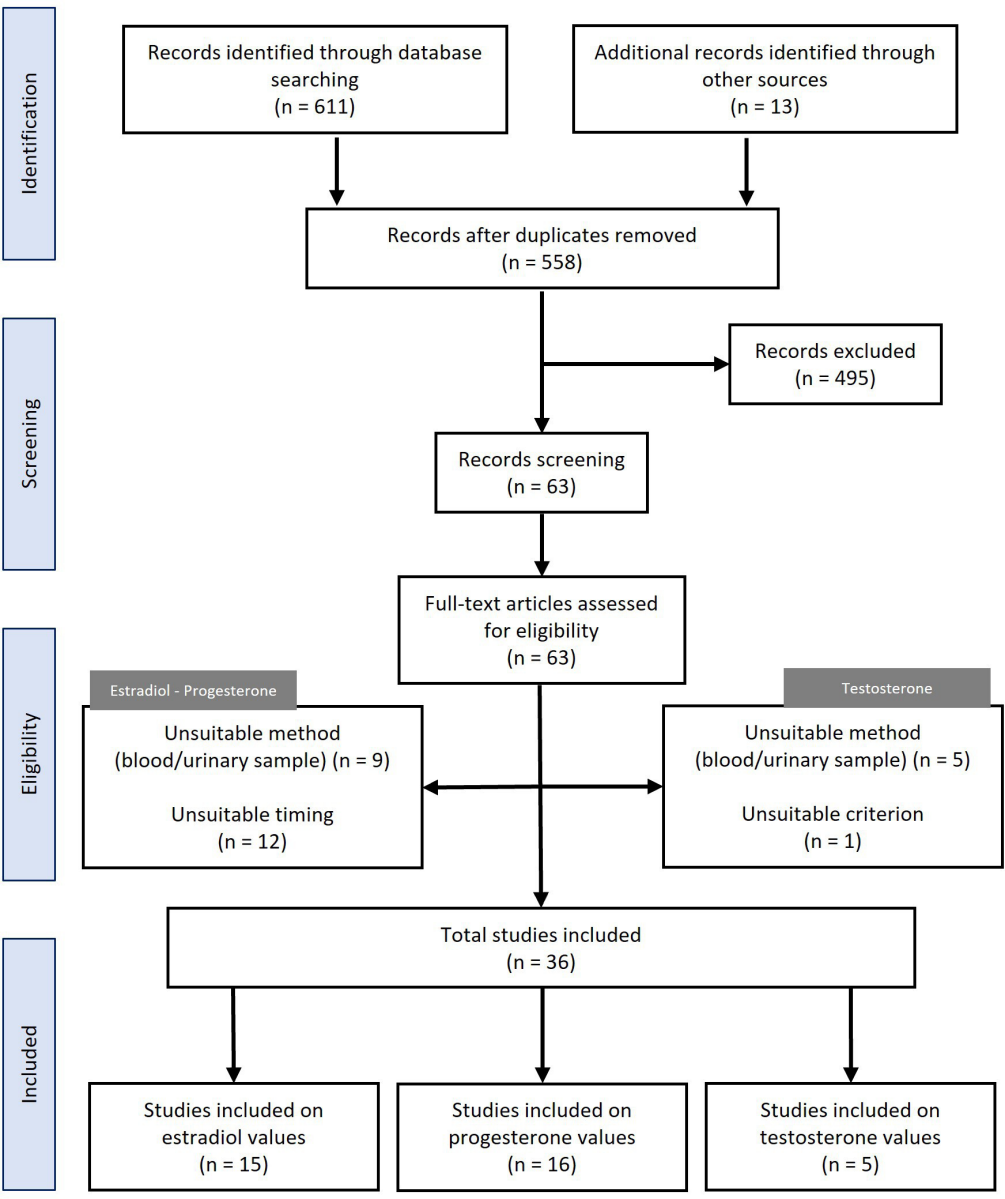
Flow chart of the bibliographic process for data on salivary hormones during the menstrual cycle in the general population.

The initial search of the three databases returned 611 articles (figure 3); 13 references from a previous bibliography were added. Based on the above exclusion criteria, 63 articles were reviewed. Among them, about salivary 17β-estradiol and progesterone, 9 studies used blood or urine, and 12 did not perform morning fasting saliva tests. Concerning salivary testosterone research, five studies used blood or urine, and one study did not perform in inclusion criterion on pre-test recommendations. All were excluded from the analysis. A total of 36 publications (online supplemental material) that investigated salivary testing 17β-estradiol (n=15), progesterone (n=16) and testosterone (n=5) in the general population during MC phases was selected for full analysis. The different methods used in these studies are detailed in online supplemental table 1. Two included studies collected saliva by stimulation (cotton or sugar-free chewing gum). Females studied in these publications were 27.03±9.1 years (median=23 years).

#### Comparison between the general population and MC athletes

**Figure 4:**
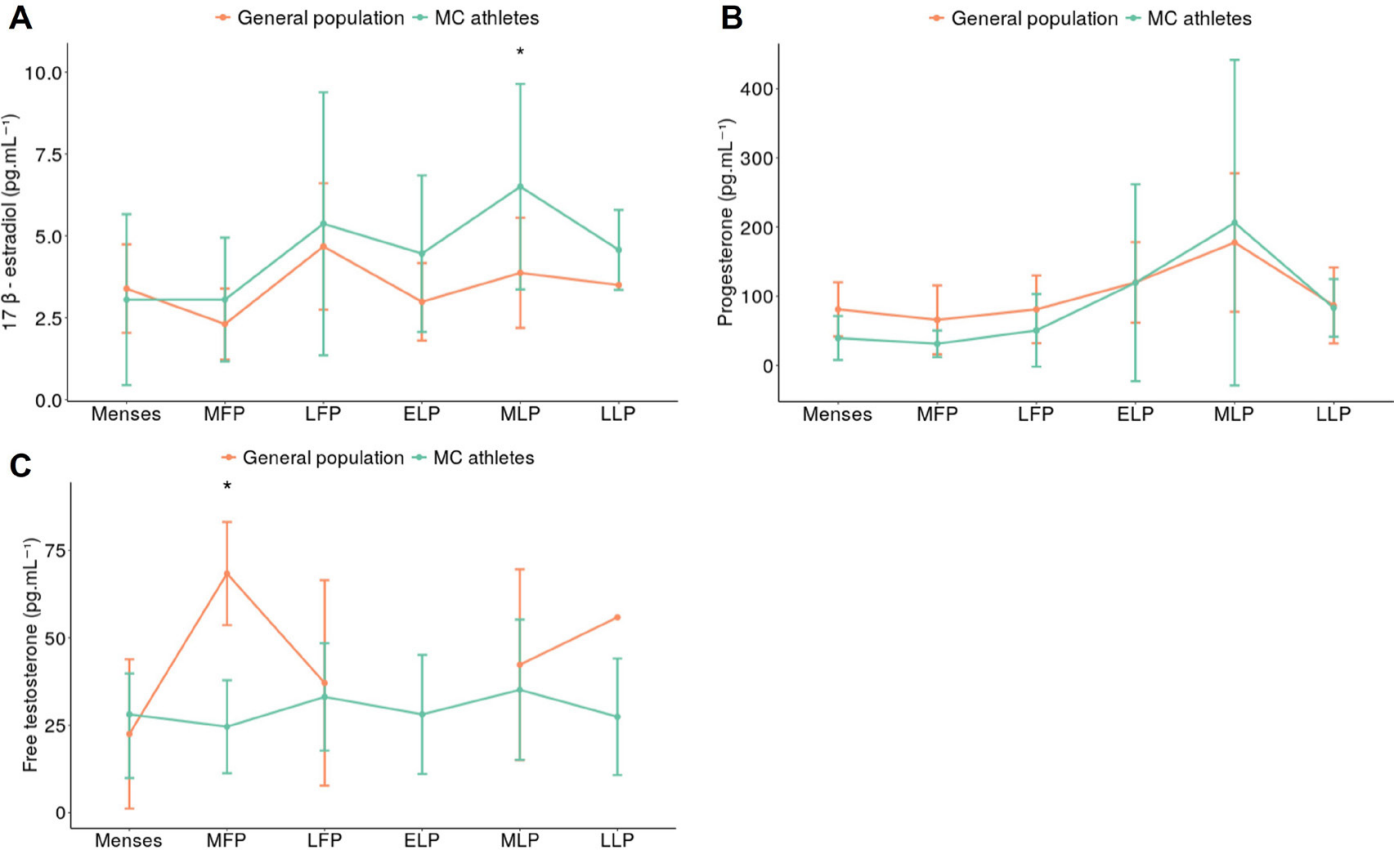
Mean and SD of salivary hormone levels (pg.mL^−1^) in the general population vs natural MC athletes during the sub-phases of the cycle. (**A**) 17β-estradiol, (**B**) Progesterone, (**C**) Free Testosterone; MFP, Mid-follicular phase; LFP, Late follicular phase; ELP, Early luteal phase; MLP, Mid-luteal phase; LLP, Late luteal phase; *p<0.05.

Comparisons of hormonal fluctuations between the general population and regular MC athletes are shown in figure 4 (*A: 17β- estradiol, B: progesterone and C: free testosterone*). No reference value for the early luteal phase (ELP) in the general population has been found. Only one reference for 17β-estradiol and free testosterone has been found for the late luteal phase (LLP).

17β-estradiol and progesterone levels significantly vary during cycles in the general population (p=0.046, p=0.01, respectively). Natural regular MC athletes have a higher saliva 17β-estradiol level than the general population in the mid-luteal phase (MLP, p=0.03).

FP progesterone levels are lower in the natural regular MC group than in the general population (p=0.04). In the mid-follicular phase (MFP), regular MC athletes also have a lower saliva-free testosterone level than the general population (p=0.02).

## Discussion

The current study aimed to establish original data of 17β-estradiol, progesterone and free testosterone for elite athletes across menstrual cycles and compare it with the general population’s values. To our knowledge, this is the first study to provide original salivary data across six MC sub-phases among elite athletes with a natural and regular MC and to analyse salivary hormonal variations in both an HC group and an irregular MC group, allowing for the assessment of hormonal variations over a standardised period.

### Saliva hormones

A double peak of 17β-estradiol was found in LFP and MLP and a peak of progesterone in MLP in regular MC athletes, as expected according to previous studies with hormonal blood samples.^44^ By comparison and following blood references, median serum progesterone concentrations were low in the FP (0.21 pmol.L^−1^). They increased in the periovulatory phase before rising sharply to a peak in the MLP (39.2 pmol.L^−1^).^45^ The coefficient of variation between these phases is greater for hormones circulating in the blood due to their concentration. Indeed, free gonadal hormones in the bloodstream enter the saliva; salivary concentrations are, therefore, lower than those in the blood. Salivary concentrations of 17β-estradiol and progesterone are typically 1–2% and 1–3% of serum concentration, respectively.^26^ There are few references in the literature concerning salivary testosterone. Generally, studies involving hormone analysis (serum, saliva, etc) analysed morning samples between 7 am and 9 am while fasting, as done in this study. For our analysis, the choice of studies on testosterone measurements in the general population was made without considering the fasting state or the time during the day. This approach was chosen due to the lack of clear data regarding salivary testosterone measurement in women while fasting, in contrast to the well-established guidelines for men.^46^ In addition, only a very small number assessed the fluctuations in salivary testosterone concentration across the MC. It is therefore important to have more information and references on salivary testosterone throughout women’s MC. Blood hormone analysis by mass spectrometry is the gold standard for method accuracy. However, analysing such dynamics while monitoring elite athletes, especially over long follow-up periods (when continuous blood serum analyses pose constraints), could represent a beneficial compromise for further study of ovulation moments. However, with further research, salivary data could become a practical alternative for long-term monitoring of elite athletes.

### 17β-estradiol and progesterone

The 17β-estradiol level doubles from the phases with the lowest concentration (menses and MFP) to the phase with the highest concentration (MLP). For natural MC, altered oestrogen levels may serve as an early indicator of MC disorders, even without changes in cycle length. Oligomenorrhoea and amenorrhoea are both associated with hypo-oestrogenic states and are diagnosed clinically.^47 48^ Our data provide reference values that were missing when testing elite athletes’ sex hormones through salivary sampling at various points in their cycle. Despite this, our approach does not allow for the precise determination of menstrual cycle phases or the detection of subtle menstrual disturbances, which could be misclassified within the regular MC group. Deviations from the values or dynamics presented in our data should raise concern when clinically assessing elite athletes. Further studies that exclude subtle cycle irregularities are necessary to refine the reference values provided here, which would be a critical step towards the early detection of subtle menstrual disturbances.

An adequate energy availability preserves oestrogen production, promoting bone health.^35^ The energy requirements of athlete training are often high in elite sports. An imbalance between energy expenditure and energy intake is known as low-energy availability, which is the centre of a large concept, the Relative Energy Deficiency in Sports (REDs).^49^ This syndrome causes the body to draw on reserves, disrupting normal metabolism through hormonal imbalances. Reduced energy availability, detected by the hypothalamus, triggers endocrine shifts, decreasing LH pulsatility and causing reproductive issues (anovulation, oligomenorrhoea or amenorrhoea).^50 51^ REDs can result in health complications, including a high injury risk rate, chronic fatigue, etc.^52^ Therefore, our values may facilitate on-field applications to identify potential hormonal changes during elite athletes’ follow-up.

Regarding serum progesterone levels, an increase from the FP to the LP is typically used to confirm ovulation.^28^ Our study found that athletes with irregular MCs^53^ did not exhibit the expected progesterone rise in the second half of the cycle. This pattern was detectable through salivary sampling in athletes with regular MCs. Therefore, monitoring progesterone levels via salivary sampling could be valuable in future research to detect irregular cycles. Additional studies are needed to establish the salivary progesterone cut-off levels indicative of ovulation.

Studying the kinetics of the 17β-estradiol and progesterone over time in long follow-up studies, where saliva sampling would be more easily implemented among elite athletes, may help detect menstrual disturbances.

### Free testosterone

We highlight the fluctuation in salivary-free testosterone during regular MC and HC. Multiple studies reported that serum-free testosterone peak occurred during mid-cycle (around ovulation).^54^,^56^ However, testosterone level variation during the rest of the cycle does not seem to show any consistent pattern. Our results show that salivary 17β-estradiol and free testosterone fluctuate similarly during the different phases of the menstrual cycle. Androgens such as testosterone are converted to estradiol by the enzyme aromatase.^57^ This conversion is mainly expressed in peripheral tissues such as the ovaries and adipose tissues. The fluctuation of testosterone during the menstrual cycle is still poorly understood. This enzymatic reaction is crucial for maintaining oestrogen levels, which could explain similar fluctuations during the menstrual cycle.^58^ In addition, testosterone secretion varies according to the type of exercise. Acute resistance exercise often leads to a short-term increase in testosterone in men,^59^ but studies investigating testosterone rise after resistance training are lacking while considering menstrual cycle phases or HC use. Further studies are needed to understand this oestrogen-androgen relationship.

### HC use in sport

The prevalence of HC use in sports varies between 20% and 70% depending on sport, country and level of competition.^60^ Athletes take contraception to avoid pregnancy but also to control and regulate their cycle and reduce the pain and symptoms.^61^ To date, very few studies carry out hormone analyses on HC users, and even fewer do so during the hormone-taking phase.

For the HC group, we observed a lower fluctuation in salivary 17β-estradiol and progesterone levels during the 28 days of pill intake/pause, as expected, due to the downregulation caused by the pills to prevent ovulation. We also observe that HC athletes have greater levels of free testosterone during the inactive phase of HC and the first week of synthetic hormones, diminishing during the next 2 weeks of hormone action. It has been shown that the dosage of ethinyl-estradiol in contraceptives leads to a reduction in the production of androgen hormones.^14^ OC lowers circulating androgen levels by directly inhibiting ovaries’ production and increasing oestrogen-induced hepatic production of SHBG. These two mechanisms result in a drop in free testosterone,^62^ an effect that may be more or less significant, depending on the type of contraception used. The dosage of synthetic hormones varies, particularly progestins, according to generations and types.^63^ The differences between HC progestins will be reflected in their potency, androgenic effects and interactions with oestrogens. There is little research comparing low and high androgenicity HC. High levels of exogenous hormones in oral HC with high androgenicity may have a greater influence than HC with low androgenicity. Further investigation of the effects of HC phases, according to the androgenicity levels, in association with performance analyses is needed.

### General population

We observed that salivary fluctuations of 17β-estradiol and progesterone of naturally menstruating athletes with a regular MC were similar to those of the general population. However, differences in 17β-estradiol levels were noted between regular athletes and the general population. Intense, acute physical exercise induces a stress response from the autonomic nervous system and the hypothalamic-pituitary-adrenal axis, potentially altering hormonal profiles.^23^ Further research is needed to understand these hormonal variations between elite athletes and the general population and investigate the potential long-term effects of such intense physical exercise according to energy intake.

### Limitations

First, this is an observational pilot study with a small sample size. It would be interesting to develop this study with a larger sample size to make the reliability and variability of the study more certain.

To compare the menstrual phases with the general population, the menstrual phases were first defined using a calendar, and the ovulation day was predicted but not measured. To increase reliability and variability, it would have been ideal to collect salivary hormones over three consecutive cycles for each athlete,^64^ but this was not done in this study. Yet, we a posteriori confirm the phases through the rise of 17β-estradiol in LFP and the progesterone rise in MLP in the regular MC group.

The original values we provided here should not be used to detect cycle phases or subtle cycle disorders but rather a large range of what is expected from salivary sampling of elite athletes’ sex hormones. Nevertheless, it is useful for showing different hormonal environments that could enlighten new research to detect subtle menstrual disturbances. For future studies with longitudinal follow-up, salivary testing is a simpler and less-invasive alternative to blood sampling.

The fact that our hormonal saliva results reproduce the results found in the general population reinforces the use of saliva testing in elite athletes when blood serum is not possible or unethical. This can also be a complementary measure to an ovulation test. A calendar-based method, in combination with urinary LH detection kits, has been recommended to help determine ovulation time in MC.^28^ Added to a calendar estimation of the follicular days, the analysis of 17β-estradiol and progesterone salivary peaks may allow us to distinguish different hormonal milieus of regular MC better. However, more recent validation studies on salivary and serum correlation should be carried out to aim to validate MC phases based solely on salivary samples. It is reasonable to assume that including athletes with potential anovulatory cycles could have contributed to increased variability and wider CIs only rather than changing the direction of the results. From this perspective, determining typical salivary values and dynamics according to the ovulatory or anovulatory cycle type may be useful.

We also acknowledge that the HC group contains different types of pills. The synthetic progestin list varies in its effects regarding oestrogenic, androgenic and progestogenic activity, possibly increasing the variability in our results.

Finally, some of the investigations used to establish data from the general population had a wider range of participant ages than our study. This range could introduce a bias in the reference values for the general population.^65^ Different hormonal measurement methods were also used in these studies. Their performance and reproducibility vary, affecting the observed mean values.^66^ Free testosterone measured in blood using an assay kit is not highly reproducible. However, salivary testosterone concentration is a better indicator of a biologically active steroid than total plasma levels, particularly under conditions where SHBG binding characteristics vary.^67^

### Clinical implications and perspectives

This is the first study to provide salivary hormonal original data on elite athletes for a six-phase MC, relying on repeated measures collected within 6 months of longitudinal follow-up, in addition to reference salivary-free testosterone values across phases of HC intake. Such results provide reference values and dynamical variations that will be useful in future studies of female athletes. These results may help researchers develop new methods of classifying the menstrual cycle phases based on salivary samples that are less invasive for athletes, enabling repeated measurements to be carried out more easily. This study could also help sports scientists identify adaptations to medical monitoring, training and recovery based on the profile and hormonal phases of athletes’ cycles. Hormonal balances and variations appear important for the onset of symptoms and training parameters. Understanding these variations may be the first step towards better approaching elite female athletes’ hormonal influences.

This topic should be studied in greater depth, with a classification of the types of contraception depending on their ethinyl-estradiol level and their progestin androgenicity. The potential relationship between hormones, well-being and training parameters should also be studied. In particular, responses to intense training loads on the days preceding a saliva test could impact the measured hormone levels.

## Conclusion

Establishing baseline values for sex hormone levels in saliva could provide foundational data for future research on elite female athletes across regular MC or combined HC. When comparing athletes under the same exposure scale, those using hormonal contraception displayed lower concentrations of 17β-estradiol, progesterone and free testosterone compared with athletes with natural regular MC, particularly after the first week of hormonal contraceptive intake. Additionally, athletes with irregular cycles did not exhibit a rise in salivary progesterone. These findings provide new tools for investigating elite athletes’ MCs or HC use through longitudinal follow-ups based on salivary samples.

## supplementary material

10.1136/bmjsem-2024-002078 online supplemental file 1

## Data Availability

Data may be obtained from a third party and are not publicly available.
