# Supplementary material for: Original salivary sex hormone data of naturally menstruating athletes and hormonal contraceptive users
Source: BMJ Open Sport Exerc Med. 2024 Nov 13;10(4):e002078. doi: 10.1136/bmjsem-2024-002078 (PMC11575394; doi:10.1136/bmjsem-2024-002078)
Supplement: online supplemental file 1 [file bmjsem-10-4-s001.pdf]

Supplementary Material

Table 1 - References of general population studies.

Reference	Title	Participants	Age (y)	Method	Hormones	Phase determination	Timing of test
[65]	Relationship between Changes in Foot Arch and Sex Differences during the Menstrual Cycle.	F = 10<br>M = 14	F = 21.1 ± 0.7<br>M = 21.9 ± 1.2	ELISA	E2<br>P4	BBT and O	EFP (3-4 days after the start of menstruation), OP (2-4 days after the ovulation day) and LP (5-10 days after the start of the high-temperature phase).<br><br>Hour : between 08:00 and 12:00
[66]	Daily cortisol awakening response and menstrual symptoms in young females.	F = 16	F = 24.0 ± 1.2	ELISA	E2<br>P4		On the 1st , 3rd , 5th , 7th , 9th, 11th, 13th, 15th, 17th, 19th, 21th, 23th, 25th, 27th , days of the cycle.<br><br>Hour: 60min post-awakening
[67]	Menstrual phase and the vascular response to	F = 18	F = 28 ± 7	Salimetric	E2	Standard O calculator (American Pregnancy Association) and confirmed with	EFP (days 1-7) and ELP (dependent upon each individual cycle length, ~ 14-19 days prior to start of menses)

	acute resistance exercise.					standard norms of salivary E2.	
[68]	Salivary estradiol and progesterone during the normal ovulatory menstrual cycle in Chinese women.	F = 10	F = 21 - 36	R	E2<br>P4	Urinary LH	Every day during the cycle.<br>Fasting
[69]	Normative study of age variation in salivary progesterone profiles.	F = 124	F = 18 - 44	R	P4		Daily LP
[70]	Normal salivary progesterone levels throughout the ovarian cycle as determined by a direct enzyme immunoassay.	F = 41	F = 27.3 ± 5.4	Direct EIA	P4	Pelvic ultrasonography	Daily fasting between 8:00 am and 10:00 am.
[71]	Non-invasive hormonal analysis for ovulation detection	F = 10	F = 22 - 33	R	E2<br>P4	BBT and MLP plasma P4 level above 16 $nmol.L^{-1}$	FP, Midcycle and LP<br>Fasting

[72]	Influence of sex, menstrual cycle, and hormonal contraceptives on egocentric navigation with or without landmarks.	F = 62 (21 in EFP, 20 in peri-OP and 21 in MLP)<br>M = 21	F = 21.2 ± 2.6<br>M = 22.2 ± 0.5	ELISA	E2<br>P4<br>T		EFP (days 1-7 days), Peri-OP (days 16-12 before their next menses) and MLP (9-3 days before next menses)
[73]	The Effects of Sex Hormonal Fluctuations during Menstrual Cycle on Cortical Excitability and Manual Dexterity (a Pilot Study).	F = 9<br>M = 9	F = 26.1 ± 5.1<br>M = 24.9 ± 5.1	EIA	E2<br>P4	Cervical fluid	FP (days 0 to 9), OP (days 12-15) and MLP (days 18 to 24).<br>Hour : between 9:00 am and 12:00 am
[74]	Menstrual cycle phase predicts women's hormonal responses to sexual stimuli.	F = 22	F = 21.9 ± 4.8	Salimetric	E2<br>P4<br>T	Puts' method (forward-counting to estimate the onset of participants' next menstrual cycles, and backward-counting to estimate o	MFP and MLP.

						O.)	
[75]	Characteristics of salivary profiles of oestradiol and progesterone in premenopausal women.	F = 56 (21 with complete ovulatory cycles in all 3 cycles, 12 with anovulation in ≥ one cycle, 5 with high E2 in all 3 cycles)	F = 42 (median)	Direct R	E2<br>P4	Detection O : P4>190 pmol.L^{-1} , cycle length >24 days, E2 values of < 44 pmol.L^{-1} during the first 10 days of cycle.	Daily morning during one cycle.
[76]	Comparison of salivary steroid profiles in naturally occurring conception and non-conception cycles.	F = 24	F = 26 – 39	R	E2<br>P4	E2 and P4 concentrations	FP (days -10 to -1) with MFP (days -10 to -6) and LFP (days -5 to -1), LP (days-0 to 5) with ELP (days 0 to 2) and MLP (days 3 to 5)
[77]	Association of menstrual cycle phase with the	F = 40	F = 25.3 ± 3.4	ELISA	E2<br>P4	E2 and P4 concentrations	EFP and MLP

	core components of empathy						Hour : between 10 am and 12 am
[78]	Sex, menstrual cycle, and hormonal contraceptives influences on global–local processing.	F = 55<br>M = 16	F = 21.2 ± 0.4<br>M = 22.2 ± 0.5	ELISA	E2<br>P4	Self-assessment of the date of onset of the last four cycles, with calculation of the average length of the last four menstrual cycles and estimation of the date of onset of the next cycle.	FP (1-7 days), OP (16-12 days before their next menses) and LP (9-3 days before their next menses)
[79]	The Effect of Menstrual Cycle Phases on Approach–Avoidance Behaviors in Women: Evidence from Conscious and Unconscious Processes.	F = 27	F = 22.78 ± 2.35	ELISA	E2<br>P4	Reverse calculation method, O LH test and hormones concentrations	EFP, LFP and MLP.
[80]	Evaluation of Salivary Electrolytes during Normal Menstrual Cycle	F = 48	F = 20 - 30	Salimetrics	E2<br>P4	Oral Body temperature, ferning	PreOP (6-12 days), OP (13-14 days) and PostOP (15-26 days).

	with Special Reference to Ovulation.					pattern and hormones levels to detect O	
[81]	Associations of endogenous 17-β-estradiol with theta amplitude and performance in semantic categorization in young women,	F = 18	F = 23.06 ± 3.35	ELISA	E2	O test	EFP, LFP and LP.<br><br>Hour : between 6:00 am and 8:00 am
[82]	Relationship between salivary progesterone, 17-hydroxyprogesterone, and cortisol levels throughout the normal menstrual cycle of healthy postmenarcheal girls.	F = 30	F = 15 – 22	R	P4	Hormone's concentrations	Every morning from day 1 of menstrual bleeding until next menses.<br><br>Hour : between 6:30 am and 8:00 am

[83]	Menstrual variation in experimental pain: correlation with gonadal hormones.	F = 32	F = 20 - 41	ELISA	E2<br>P4<br>T	Self-reported usual cycle phase and length, P4 levels.	Day 1, 4, 14 and 22 (± 1 day) of the menstrual cycle.
[84]	Natural Variation in Testosterone is Associated With Hypoalgesia in Healthy Women.	F = 40	F = 18 – 53	Salimetrics	E2<br>P4<br>T	Urinary LH test	MFP, OP and LLP.
[85]	The role of sex hormones, oral contraceptive use, and its parameters on visuospatial abilities, verbal fluency, and verbal memory.	F = 100<br>M = 47	F = 23 – 35<br>M = 23 – 35	Liquid chromatography-tandem mass spectrometry	T	Hormone's concentrations	EFP and preOP

F = Female, M= men, E2 = salivary 17β-estradiol, P4 = salivary progesterone, T = salivary testosterone, FP = follicular phase, LP = luteal phase, EFP = early follicular phase, MFP =mid-follicular phase, LFP = late follicular phase, ELP = early luteal phase, MLP = mid-luteal phase, LLP = late luteal phase, OP = ovulatory phase, PreOP = preovulatory phase, PostOP= postovulatory phase, O = ovulation, BBT = basal body temperature, ELISA = Enzyme-linked-immunosorbent assay, R = Radioimmunoassays, EIA = enzymeimmunoassay, LH = luteininzing hormone.

Table 2 - Participant characteristics of MC group and Hormonal group. Data presented as mean ± SD.

Groups	N	Age (y)	Weight(kg)	Height (cm)	Contraception
**ALL**	54	24.5 ± 4.6	63.7 ± 9.7	170.10 ± 8.5	-
**MC group**	20	25.0 ± 4.2	65.9 ± 11.0	171.5 ± 10.0	-
IUDs copper	2	26.2 ± 0.9	63.5 ± 9.1	174.0 ± 1.4	-
**HC group**	34	24.2 ±5.2	62.4 ± 8.7	169.2 ± 7.4	
Monophasic	16	23.2 ± 2.7	63.1 ± 8.7	169.9 ± 6.0	Optidril 30®, Minidril®, Optilova 20®, Zoely®, Leeloo®, Yaz®
Biphasic	2	24.1 ± 1.9	61.5 ± 17.6	172.7 ± 6.0	Adepal®
Triphasic	5	28.7 ± 6.3	62.2 ± 6.9	165.8 ± 8.8	Daily ge®
Progesterone-only	1	23.5 ± 6.7	61.6 ± 7.5	170.3 ± 6.4	Slinda®
Continuous pill	6	22.8 ± 3.0	59.5 ± 11.1	166.1 ± 11.2	Optimizette®
IUDs hormonal	3	26.1 ± 4.7	64.6 ± 8.7	173.6 ± 4.5	Mirena®, Kyleena®
Ring	1	20.9 ± 0.0	69.0 ± 0.0	173.0 ± 0.0	Nuvaring®

MC = Menstrual cycle ; HC = Hormonal contraception; IUD = Intrauterine Device

Table 3 - Details of hormonal contraceptive

Brand	Synthetic estrogens	Synthetic progestins	Other
Optidril 30®	Ethinylestradiol : 0.03 mg	Levonorgestrel : 0.15 mg	
Minidril®	Ethinylestradiol : 0.03 mg	Levonorgestrel : 0.15 mg	
Optilova 20®	Ethinylestradiol : 0.02 mg	Levonorgestrel : 0.1 mg	
Leelo ®	Ethinylestradiol : 0.02 mg	Levonorgestrel : 0.1 mg	
Yaz®	Ethinylestradiol : 0.02 mg	Drospirenone : 3 mg	
Zoely®	-	Nomegestrol acetate : 2.5 mg	Estradiol : 1.5 mg
Adepal®	Ethinylestradiol : 0.03 mg (7 days)<br>0.04 mg (14 days)	Levonorgestrel : 0.15 mg (7 days)<br>0.2 mg (14 days)	
Daily ge®	Ethinylestradiol : 0.03 mg (6 days)<br>0.04 mg (5 days)<br>0.03 mg (10 days)	Levonorgestrel : 0.05 mg (6 days)<br>0.075 mg (5 days)<br>0.125 mg (10 days)	
Slinda®	-	Drospirenone : 4 mg	
Optimizette®	-	Desogestrel : 0.075 mg	
Mirena®	-	Levonorgestrel : 52 mg	
Kyleena®	-	Levonorgestrel : 19.5 mg	
Nuvaring®	Ethinylestradiol : 2.7 mg	Etonogestrel: 11.7 mg	

Salivary test and Luminescent immunoassay method

Salivary 17 β*-estradiol*

The calibration was established by use of 17β-estradiol ranging from 0 to 64 pg.mL^{-1}. Cross reactivities (Abraham method) of the antiserum were measured against various compounds : Estrone –14%; Deoxycortisol – 0.58%; Estriol – 0.5%; Fulvestrant – 0.42%; Estrone-3-sulfate – 0.26%; and other substances<0.05. Analytical sensitivity was calculated from the mean of the relative luminescence units of the zero calibrator minus 3 standard deviations of 30 replicate analyses. The lowest detectable level that could be distinguished from the zero standard is 0.3 pg.mL^{-1}. Functional sensitivity was determined from the inter-assay variation coefficient of very low concentrations in saliva samples. The lowest 17β-estradiol concentration that could be measured with a coefficient of variation below 20% is 0.9 pg.mL^{-1}. The mean intra-assay precision of twenty runs with a panel of 3 human saliva samples in the mean range 1.6 – 39.6 pg.mL^{-1} was found to be 10%, and a range of 7.2 - 13.3 %. The mean inter-assay precision of 5 runs with a panel of 10 human saliva samples in the mean range 1.82 - 36.16 pg.mL^{-1} was found to be 6.4 %, and a range of 4.0 - 11.6 %. The mean inter-lot precision of 3 saliva samples in the mean range 1.88 – 40.00 pg.mL^{-1} was found to be 12.8 %, and a range of 8.1 - 25.1 %.

Salivary progesterone

The calibration was established by use of progesterone ranging from 2.6 to 1000 pg.mL^{-1}. Cross reactivities (Abraham method) of the antiserum were measured against various compounds: 17α-OH-Progesterone – 1.84%; 6α-Methyl-17α-OH-Progesterone – 1.41%; Pregnenolone – 0.41%; Desoxy-Corticosterone– 0.28%; Androsterone Sulfate– 0.25%; Androstenedione – 0.20% ; Androsterone - 0.20% ; DHEA-S – 0.11% and Corticosterone – 0.06. Analytical sensitivity was calculated from the mean of the relative luminescence units of the zero calibrator minus standard deviations of 30 replicate analyses. The lowest detectable level that could be distinguished from the zero standard is 2.4 pg.mL^{-1}. Functional sensitivity was determined from the inter-assay variation coefficient of very low concentrations in saliva samples. The lowest progesterone concentration that could be measured with a coefficient of variation below 20% is 8.9 pg.mL^{-1}.. The mean intra-assay precision of twenty runs with a panel of 3 human saliva samples in the mean range 1.6 – 39.6 pg.mL^{-1} was found to be 10%, and a range of 7.2 -13.3 %. The mean inter-assay precision of 5 runs with a panel of 10 human saliva samples in the mean range 1.82 - 36.16 pg.mL^{-1} was found to be 6.4 %, and a range of 4.0 - 11.6 %. The mean inter-lot precision of three saliva samples in the mean range 1.88 – 40.00 pg.mL^{-1} was found to be 12.8 %, and a range of 8.1 - 25.1 %. The mean precision of 10 saliva samples was found to be 7.7% (a range of 4.3% - 14.2%) and 9.5% (a range of 6.0% - 15.2%) for intra-assay and inter-assay variation.

Salivary free testosterone

The calibration was established by use of free testosterone ranging from 0 to 760 pg.mL^{-1}. The detection range of the luminescent immunoassay encompasses the physiological range of testosterone concentrations in saliva and in 1:40 diluted serum samples. Cross reactivities (Abraham method) of the antiserum were measured against various compounds: 11β-hydroxytestosterone – 8.7%; 11α-hydroxytestosterone – 3.2%; 5α-dihydrotestosterone – 1.9%; androstendione – 0.8%; methyltestosterone – 0.44%; DHEAS – 0.07%; testosterone sulfate : 0.04%, progesterone : 0.03% and other steroids < 0.01. Analytical sensitivity was calculated from the mean of the relative luminescence units of the zero calibrator minus 2 standard deviations of 20 replicate analyses. The lowest detectable level that could be distinguished from the zero standard is 1.8 pg.mL^{-1}.. The lowest free testosterone concentration that could be measured with a coefficient of variation below 20% is 3.9 pg.mL^{-1}. The mean intra-assay precision of three saliva samples in the range 12.8 – 223.9 pg.mL^{-1} was found to be 9.1%, and a range of 4.0 - 16.7 %. The mean inter-assay precision of ten saliva samples in the range 5.54 - 557.3 pg.mL^{-1} was found to be 4.7 %, and a range of 2.90 - 6.96 %. The mean inter-lot precision of three saliva samples in the range 12.8 – 223.9 pg.mL^{-1} was found to be 9.2%, and a range of 0.0 - 16.0 %.
